# Antioxidant properties and phenolic profiling by UPLC-QTOF-MS of Ajwah, Safawy and Sukkari cultivars of date palm

**DOI:** 10.1016/j.bbrep.2021.100909

**Published:** 2021-01-18

**Authors:** S.M. Neamul Kabir Zihad, Shaikh Jamal Uddin, Nazifa Sifat, Farhana Lovely, Razina Rouf, Jamil A. Shilpi, Bassem Yousef Sheikh, Ulf Göransson

**Affiliations:** a Pharmacy Discipline, Life Science School, Khulna University, Khulna, 9208, Bangladesh; b Department of Pharmacy, Faculty of Life Science, Bangabandhu Sheikh Mujibur Rahman Science and Technology University, Gopalganj, 8100, Bangladesh; c College of Medicine, Taibah University, PO Box 456, Almadinah Almunawarah, 41411, Saudi Arabia; d Division of Pharmacognosy, Uppsala University, Biomedical Center, Uppsala, SE, 75123, Sweden; e Department of Medicinal Chemistry, Uppsala University, Biomedical Center, Box 574, Uppsala, SE, 75123, Sweden

**Keywords:** Date palm, Antioxidant activity, Free radical, Phenolics, Flavonoids

## Abstract

Date palm (*P. dactylifera*) plays a vital role in ethnomedicinal practices in several parts of the world. There are over 2000 cultivars of date palm that differ in chemical composition and extent of bioactivity. The present study was undertaken to comparatively evaluate the antioxidant potential of three cultivars of date palm (Ajwah, Safawy and Sukkari) from Saudi Arabia and analyze their phenolic constituents in order to draw a rationale for their activity. Antioxidant activities of the date cultivars were evaluated by different quantitative methods including 2,2-diphenyl-1-picrylhydrazyl (DPPH) and hydroxyl radical scavenging assay, total antioxidant capacity, reducing power, total phenolic (TPC), flavonoid (TFC) and tannin content (TTC), while qualitative phenolic composition was determined using ultra performance liquid chromatography coupled to quadropole time of flight mass spectrometry (UPLC-QTOF-MS). All the three date extracts showed good DPPH radical scavenging (IC_50_ 103–177 μg/mL) and hydroxyl radical scavenging (IC_50_ 1.1–1.55 mg/mL) activity and total antioxidant capacity (IC_50_ 87–192 μg/mL). The reducing power was also comparable to that of ascorbic acid, used as standard in above experiments. All the three samples contain significant amount of major antioxidant components (phenolic, flavonoid and tannin) that successfully correlates with the results of radical scavenging assays. UPLC-QTOF-MS revealed a total of 22 compounds in these date cultivars classified into common phenolics, flavonoids, sterols and phytoestrogens. Significant variation in the degree of antioxidant activity of these three date cultivars can be attributed to the difference in the content and composition of phenolic compounds.

## Highlights

• Ajwah, Safawy and Sukkari date palm possess significant amount of natural antioxidant components.
• Dietary intake of Ajwah, Safawy and Sukkari date palm can prevent a number of oxidative damage mediated diseases.
• The common phenolics, flavonoids, sterols and phytoestrogens of date palm might contributed in the prevention of diseases.

## 1 Introduction

Fruits have been a major part of human diet since antiquity and it is considered very important and beneficial for health as they serve both as nutritional source and natural mean of health promotion [1]. Numerous studies have been conducted to evaluate medicinal properties and health benefits of fruits with the outcome that they can prevent a number of chronic diseases including coronary heart disease [2,3], cancer [4], atherosclerosis [5], neurodegenerative disorders [6,7], and inflammation [8]. Studies suggest that the polyphenols, especially flavonoids can be partly attributed to such activities of fruits [9,10]. Nowadays, inclusion of fruit in our daily diet is recommended by different regulatory bodies for maninaing disease free sound health.

The fruit of *Phoenix dactylifera,* commonly known as date palm, is a very popular fruit that grows in the arid regions with extreme environmental conditions. It is native to Africa and Persian Gulf region but the origin is not certain. Iraq, Egypt, Saudi Arabia, Tunisia, Algeria, UAE, Oman, Libya, Pakistan and Sudan are the top producers of date palm in the world [11]. At present around 2000 cultivars of date palm are grown all over the world [12]. Date palm plays an important social, environmental and economic role in these regions as principle financial and food source. Use of date palm appeared in various ancient societies (e.g., Egyptian, Roman) and religious traditions (e.g., Jewish, Christian and Islamic) [13]. The date fruits are a very good source of rapid energy and highly rich in nutrients [14]. Apart from the rich nutritional value, dates are used in ethnobotanical practices for a number of ailments including liver disorders, diabetes, constipation, diarrhea, asthma, bronchitis, respiratory disorders and headache [15]. Several studies have scientifically demonstrated its anti-inflammatory [16], antioxidant [1,17,18], antihyperlipidemic [19], antimutagenic [17], anticancer [20], antiviral [21], antifungal [22], gastroprotective [23], hepatoprotective [24,25], nephroprotective [26], antihaemolytic [27], immunostimulating [28], gonadotropic [29], neuropharmacological and analgesic [15] activities. Dates are potential source of a number of bioactive phytochemicals. Date are reported to possess simple pheolic acids (gallic acid, vanillic acid, syringic acid), carotenoids (lutein, β-carotene), flavonoids and their derivatives (catechin, *epi*-catechin, quercetin, apigenin) [30], phytosterols (cholesterol, campesterol, β-sitosterol) [31], phenylpropanoids (caffeic acid, 5-*O*-caffeoylshikimic acid, ferulic acid etc) [1] and anthocyanins [18]. The chemical composition of dates varies among different cultivars, soil condition, agronomic practices and ripening stages [32,33].

Ajwah, Safawy and Sukkari are three popular date cultivars growing in Saudi Arabia. Saudi Arabia is the second highest producer of date palm and grow around 300 cultivars [34]. Among the three cultivars, Sukkari is the best-selling date in Saudi Arabia having a golden brown color and firm texture. Safawy, a dark brown date, is another common date cultivar in Saudi Arabia which is characterized by its high productivity. Relatively small, round shaped and black colored Ajwah is the most prolific cultivar of date palm in Saudi Arabia as it is mentioned in the prophetic medicine.

Present investigation was undertaken to evaluate the antioxidant activity of these date cultivars and draw a comparative picture. Furthermore, UPLC-QTOF-MS was done for comparative analysis of the phenolic composition of these date cultivars and draw a rationale for their activity.

## 2 Materials and methods

### 2.1 Plant material and extraction

**Fig. 1:**
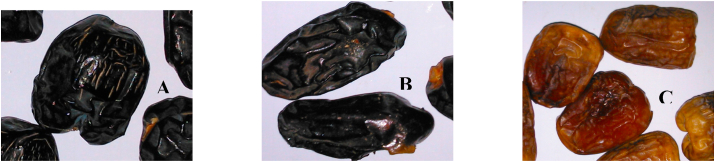
Images of collected Date palms. A: Ajwah; B: Safawy; C: Sukkari.

The dried ripe dates were purchased from the local date market in Al Madinah, Saudi Arabia and identified by taxonomists at Bangladesh National Herbarium where a voucher specimen (DACB 41158) has been submitted for future reference. The dried dates were mashed with the help of a blender and soaked in ethanol with periodic sonication. The extracts were filtered and dried using rotary vacuum evaporator at 45 °C to get semisolid masses. The extracts were further freeze dried to get the dried extracts. For easy identification, pictures of the date are provided along with the article (Fig. 1).

### 2.2 Chemicals

Acetonitrile, formic acid, tannic acid, Folin-Ciocalteu reagent, 2,2-diphenyl-1-picrylhydrazyl (DPPH), sodium carbonate, sodium nitrite, aluminum trichloride, sodium hydroxide, potassium ferricyanide, dibasic sodium phosphate, monobasic sodium phosphate, trichloroacetic acid, ferric chloride, ammonium molybdate, sodium phosphate, sulfuric acid, ascorbic acid, hydrogen peroxide, gallic acid and quercetin were purchased from Sigma–Aldrich (St. Louis, MO, USA).

### 2.3 DPPH free radical scavenging assay

DPPH free radical scavenging activity of the date extracts were determined by the method of Brand-Williams et al. (1995) [35]. Aliquots (50 μl) of serially diluted (500–0.98 μg/mL) extract solutions in methanol were mixed with 5 mL of DPPH solution (40 μg/mL) in methanol. The reaction mixture was vortexed thoroughly and left in the dark at room temperature for 30 min. Then the absorbance of the mixture was measured at 517 nm in a UV spectrophotometer (Shimadzu 2000). Ascorbic acid was used as positive control and percent inhibition was calculated using the following equation:

$$\text{\%}\cdot \text{Inhibition}\cdot =\cdot \left[1-\cdot \left({\text{Abs}}_{\text{sample}}\cdot \text{/}\cdot {\text{Abs}}_{\text{control}}\right)\right]\cdot \text{×}\cdot 100\cdot \text{\%}$$

where Abs_sample_ is the absorbance of the extract/standard solution in methanol while Abs_control_ is the absorbance of the DPPH solution in methanol.

### 2.4 Hydroxyl radical scavenging ability

Hydroxyl radical scavenging capacity of the date extracts were evaluated as per Smirnoff and Cumbes (1989) with slight modification [36]. Briefly, 1 mL of ferrous sulfate solution (1.5 mM) was mixed with 1 mL extract or standard (ascorbic acid) solution of different concentrations (0.5–2.5 mg/mL) followed by the addition of the reaction mixture (0.7 mL of 6 mM hydrogen peroxide and 0.3 mL of 2 mM sodium salicylate) which was incubated for 1 h at 37 °C. The absorbance of the hydroxylated salicylate complex was measured at 562 nm. The percent scavenging capacity was calculated as:

$$\text{Scavenging}\cdot \text{activity}\cdot =\cdot \left[1-\cdot \left({\text{A}}_{1}\cdot -\cdot {\text{A}}_{2}\right)\cdot \text{/}\cdot {\text{A}}_{\text{0}}\right]\cdot \text{×}\cdot 100$$

where A_0_ is absorbance of the control (without extract) and A_1_ is the absorbance in presence of the extract, A_2_ is the absorbance without sodium salicylate.

### 2.5 Total antioxidant capacity

The modified method described by Yang et al. (2014) was used for this assay [37]. In this test, 0.3 mL solution of extract or standard (ascorbic acid) was mixed with 3 mL of reagent mixture (4 mM ammonium molybdate, 0.6 M sulfuric acid and 28 mM trisodium phosphate). This reaction mixture was incubated at 90 °C for 90 min followed by cooling to room temperature. The absorbance of the reaction mixture was measured at 695 nm. The IC_50_ values were calculated by comparison with the absorbance of blank preparation.

### 2.6 Reducing power assay

Reducing power of the dates was measured through the reduction of Fe^3+^ as per Hazra et al. (2008) with some modifications [38]. Briefly, 1 mL solution of extract or standard (ascorbic acid) was mixed with 2.5 mL of phosphate buffer (0.2 M, pH 6.6) and 2.5 mL of potassium ferricyanide (1%). The reaction mixture was incubated for 20 min at 50 °C followed by the addition of 2.5 mL of trichloroacetic acid (10%). The upper portion of the mixture (2.5 mL) was separated after centrifugation. Then 0.5 mL of distilled water, and 0.5 mL of ferric chloride solution (0.1%) was added to it and kept at room temperature for 10 min. The absorbance was measured at 700 nm against a blank solution.

### 2.7 Phytochemical estimation of antioxidant components (total phenolic, flavonoid and tannin content)

Total phenolic content (TPC), total flavonoid content (TFC) and total tannin content (TTC) of the date extracts were determined using Folin–Ciocalteu's reagent using gallic acid, quercetin and tannic acid as standards, respectively [38,39].’ The results of TPC, TFC and TTC contents were calculated from standard curves and expressed as gallic acid equivalent (mg GAE/g), quercetin equivalent (mg QE/g) and tannic acid equivalent (mg TAE/g) respectively.

### 2.8 Analysis of phenolic composition using UPLC-QTOF-MS

Chemical composition of the three date cultivars was analyzed by chromatographic separation and mass spectrometry. Chromatographic separation was done using a Waters Nano Acquity UPLC system equipped with a reversed phase analytical column of 75 μm i.d. × 150 mm, 1.7 μm particle size (Waters QTOF Micro fed by a Waters NanoAcquity UPLC, Waters Corporation, Milford, USA). Column oven temperature was maintained at 35 °C and flow rate was set at 0.3 μL/min throughout the experiment. Water and acetonitrile, each containing 0.1% formic acid, were used as mobile phase A and B, respectively. The injection volume was 2 μL with a run time of 75 min. The linear gradient program was set as follows: 0 min, 95% A; 0–70 min, 5–95% B; 70–75 min, 95% B. The UPLC was hyphenated to a Waters QTOF micro mass spectrometer operated in positive ion mode. The nebulization gas (nitrogen) was set to 500 l/h at 350 °C; the cone gas (nitrogen) was set to 50 l/h, and the source temperature to 100 °C. The capillary and cone voltages were set to 3100 and 35 V, respectively. The MCP detector voltage was set to 4300 V. The QTOF micro MS acquisition rate was set at 0.5 s with inter scan delay of 0.1 s. Argon was used as collision and spray gas. Full scan data acquisition was performed, scanning from *m/z* 1 to 1000.

### 2.9 Statistical analysis

Results are expressed as mean ± SD from three separate observations. Linear regression equations were constructed and correlation coefficient (R) values were calculated using Microsoft Excel 2007 for correlation study between the IC_50_ values and antioxidant components. One-way ANOVA followed by Newman- Keuls post hoc test was done for statistical analysis. Graphs were prepared using Graph Pad Prism 5 software.

## 3 Results and discussion

### 3.1 DPPH free radical scavenging assay

Oxidative stress, one of the main reasons behind different pathological conditions of human body, is originated from an imbalance between body's intrinsic defense and generation of free radicals [40]. These unstable free radicals and reactive oxygen species (ROS) originate either from normal metabolic processes in human body or from external sources (X-rays, industrial chemicals, cigarette smoking, air pollutants etc.) [41] and damage cellular macromolecules like protein, lipid and DNA through electron pairing [42] resulting in a number of pathological conditions including degenerative diseases, cancer and inflammatory diseases [43]. In this study, we evaluated the free radical scavenging activity of the date palms by DPPH free radical scavenging assay. DPPH based free radical scavenging method is on of the most sensitive and easy way to examine the antioxidant activity of the natural products with the advantage of being unaffected by side reactions [44]. This N-centered radical possesses an unpaired valence electron on its nitrogen bridge that shows absorbance within the range between 515 and 517 nm and reduction in this absorbance due to radical neutralization by antioxidant species is the basis of this assay [45]. The results of DPPH free radical scavenging assay are presented in Table 1. Among the three date cultivars, Safawy was found to be the strongest scavenger of DPPH free radical. The order of activity stands as Safawy > Ajwah > Sukkari (IC_50_ 104, 125 and 177 μg/mL, respectively) and all the three extracts showing good scavenging activity (IC_50_ < 1 mg/mL) when compared to the positive control, i.e., ascorbic acid (IC_50_ 12.09 μg/mL). It can be postulated from the results that the extracts have good proton donating capacity and can be classified as primary antioxidants. In our previous study the phenolic profile of Safawy and Ajwah dates were found to be superior to that of Sukkari which is in accordance with our present finding [15].

**Table 1:** Free radical scavenging activity of the date cultivars.

Sample	IC_50_ values		
	DPPH scavenging (μg/mL)	Hydroxyl radical scavenging (mg/mL)	Total antioxidant activity (μg/mL)
Ajwah	125.16 ± 6.72*	1.1 ± 0.04*	119.14 ± 5.35*
Safawy	103.93 ± 8.18*	1.22 ± 0.05*	87.60 ± 4.7*
Sukkari	176.9 ± 11.58**	1.55 ± 0.08**	192.66 ± 6.6**
Ascorbic acid	12.09 ± 0.22**	0.55 ± 0.05**	12.30 ± 0.06**

Results are expressed as Mean ± SD where, n = 3. Superscript denote that values are significantly different from each other, where *<0.05 and **<0.005.

### 3.2 Hydroxyl radical scavenging ability

Hydroxyl radical is the major reactive oxygen species that initiates polymerization, fragmentation and auto-oxidation of biological macromolecules [46]. The dates were evaluated for their hydroxyl radical scavenging activity through the inhibition of hydroxyl radical generated from ferrous sulfate and hydrogen peroxide systems and the results are shown in Table 1. IC_50_ of the date extracts were calculated from the % inhibition vs log concentration curve. Ajwah date cultivar showed more potent hydroxyl radical scavenging capacity than the other two cultivars. Scavenging activity of the samples was in the following order: Ajwah > Safawy > Sukkari (IC_50_ 1.1, 1.22 and 1.55 mg/mL) and the results were comparable to that of ascorbic acid (0.55 mg/mL). It can be concluded from this study that these dates can be beneficial to reverse the detrimental effects of hydroxyl radical in our body, provided that necessary in vivo studies are done to approve our hypothesis.

### 3.3 Total antioxidant capacity

Total antioxidant capacity is a sensitive way to evaluate antioxidant activity of plant extracts which is, to some extent is contributed by the phenolics, flavonoids and other reducing compounds present in the extract [47]. In this project, the total antioxidant activity of the date extracts were investigated by the phosphomolybdenum method, based on the reduction of Mo(VI) to Mo(V) (a green phosphate complex with a maximal absorption) which is measured spectrophotometrically at 695 nm and the results are expressed in terms of IC_50_ values (Table 1). Total antioxidant capacity of the date cultivars can be ranked as Safawy > Ajwah > Sukkari (IC_50_ 88, 119 and 193 μg/mL) while IC_50_ of standard ascorbic acid was 12.30 μg/mL. Our previous study [15] as well as present study finds considerable amount of polyphenols in the date extracts and the difference in their amounts can be directly linked to the observed activity since the total antioxidant capacity is directly proportional to the amount of polyphenols present in the extract [48].

### 3.4 Reducing power assay

**Fig. 2:**
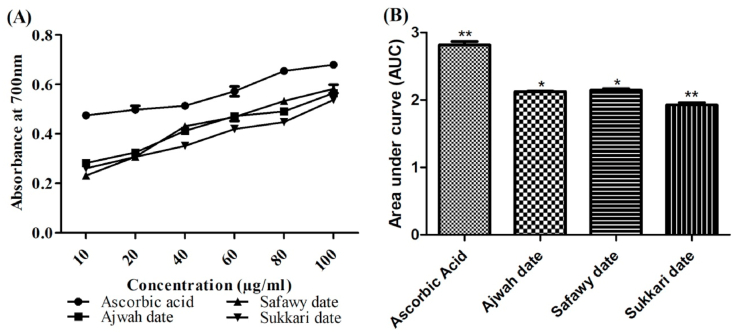
(A) Absorbance and (B) Area under curve of the date extracts and ascorbic acid in reducing power assay. Each value represents mean ± SD (n = 3). *<0.05 and **<0.005 denote that values are significantly different from each other.

The activity of antioxidants is usually attributed to a number of mechanisms including reducing capacity, inhibition of chain initiation, radical scavenging and decomposition of peroxides [49]. Thus, reducing power serves as an important index of antioxidant activity of plant extracts. In this study, the date extracts were evaluated for their reducing power through the transformation of Fe(III) to Fe(II) that is contributed through the reducing ability of the extracts i.e. ability to donate electrons in the transformation of Fe(III) to Fe(II). Fig. 2(A) shows the absorbance of different concentrations of the extracts recorded at 700 nm which reveals a direct correlation between concentration and reducing power for all the three date extracts. Safawy exhibited the maximum absorbance of 0.582 at a concentration of 0.1 mg/mL while Sukkari exhibited the minimum absorbance of 0.565 at 0.1 mg/mL. Results of areas under curve (AUC) demonstrate that Safawy possesses higher reducing power (AUC 2.15) than Ajwah (AUC 2.12) and Sukkari (AUC 1.93) date (Fig. 2(B)). When compared to the positive control, i.e., ascorbic acid (absorbance 0.68 and 0.475 at 0.1 and 0.01 mg/mL, respectively; AUC 2.82), all the three date cultivars seems to possess moderate reducing activity meaning they may not contain high amount of reductants.

### 3.5 Phytochemical estimation of antioxidant components (total phenolic, flavonoid and tannin content)

Polyphenols are naturally occurring secondary metabolites comprising of various classes of compounds (phenolic acids, flavonoids, phytoestrogens etc.) found in abundant in fruits, vegetables, cereals and beverages [50]. There is strong evidence that consumption of foods containing high amount of polyphenols can be effective against a number of diseases including asthma, infection, aging, heart diseases and cancer [50,51]. Phenolic compounds exert strong antioxidant activity due to the presence of hydroxyl groups and conjugated ring structure that contribute to their ability to scavenge free radicals. Among different classes of polyphenols, flavonoids are the most potent scavengers against most oxidizing molecules [52]. Another group of compounds, directly linked with antioxidant activity, is the tannins that are also widely distributed in almost every plant and have a great impact on human health [53]. The three date cultivars were found to be rich in these groups of phytochemicals that can render strong antioxidant potential. Results revealed that Safawy date contains the highest phenolic content (101.66 mg GAE/g) while Sukkari date has the lowest amount (39.01 mg GAE/g) among the three date extracts. The flavonoid content followed the same trend, i.e., Safawy > Ajwah > Sukkari (78.6, 71.6 and 61.0 mg QE⁄g, respectively). This order of phenolic and flavonoid content in the date extracts are in harmony with the results found in free radical scavenging assays. However, in terms of tannin content, Sukkari was found to be superior than Safawy and Ajwah date cultivars (Table 2). The results found in this study deviate by a significant and noticeable amount from previously published studies regarding the antioxidant potential of dates [1,17,18] which could be due to a number of factors involved such as difference in cultivars, soil conditions, ripening stages, storage conditions, extraction process and method of analysis.

**Table 2:** Total phenolic, flavonoid and tannin contents of selected date cultivars.

Sample	TPC	TFC	TTC
	mg GAE/g of dry extract	mg QE/g of dry extract	mg TAE/g of dry extract
Ajwah	93.37 ± 1.67*	71.6 ± 4.98	37.61 ± 0.33*
Safawy	101.66 ± 1.68*	78.63 ± 1.66	38.09 ± 0.33
Sukkari	39.01 ± 3.34**	61.03 ± 0.09*	40.56 ± 0.49**

Results are expressed as Mean ± SD where, n = 3. Superscript denote that values are significantly different from each other, where *<0.05 and **<0.005.

Furthermore, we analyzed the linear regression equations to study the association of these antioxidant components with the results found in antioxidant assay and the values of correlation coefficient (R) are given in Table 3. Nine R values ranging from 0.789 to 0.994 were observed when the IC_50_ values from the three radical scavenging assays were plotted against TPC, TFC and TTC. These high values of R suggest that the antioxidant activities of the three date cultivars might be a result of their phenolic, flavonoid and tannin content since these phytoconstituents are the main antioxidant principles found in natural sources.

**Table 3:** R values of correlation study of IC_50_ of different antioxidant assays with TPC, TFC and TTC.

Antioxidant assay	R values		
	Total phenolic content	Total flavonoid content	Total tannin content
DPPH radical scavenging assay	0.986	0.992	0.906
Hydroxyl radical scavenging assay	0.930	0.789	0.994
Total antioxidant capacity	0.984	0.993	0.901

### 3.6 Analysis of phenolic composition using UPLC-QTOF-MS

**Fig. 3:**
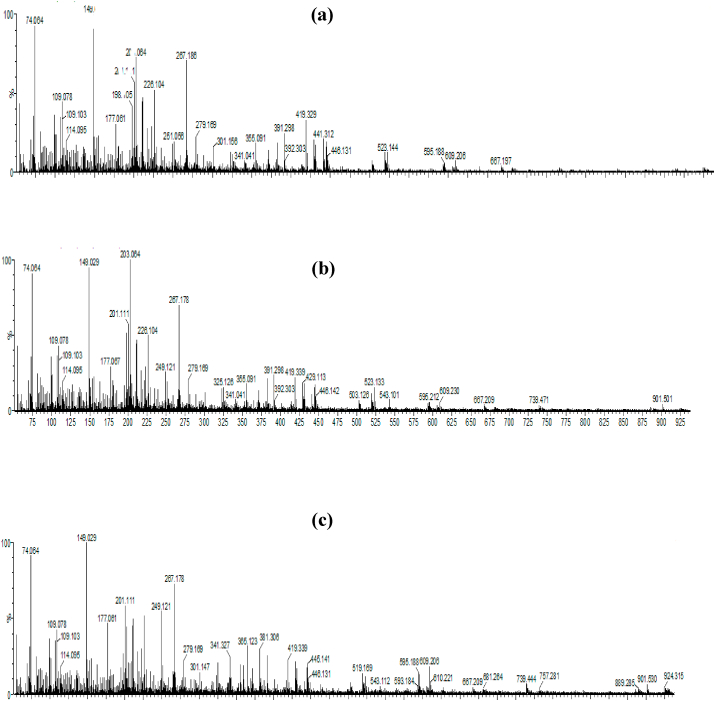
Total ion chromatogram of UPLC-QTOF-MS study of date extracts. a: Ajwah; b: Safawy; c: Sukkari.

**Fig. 4:**
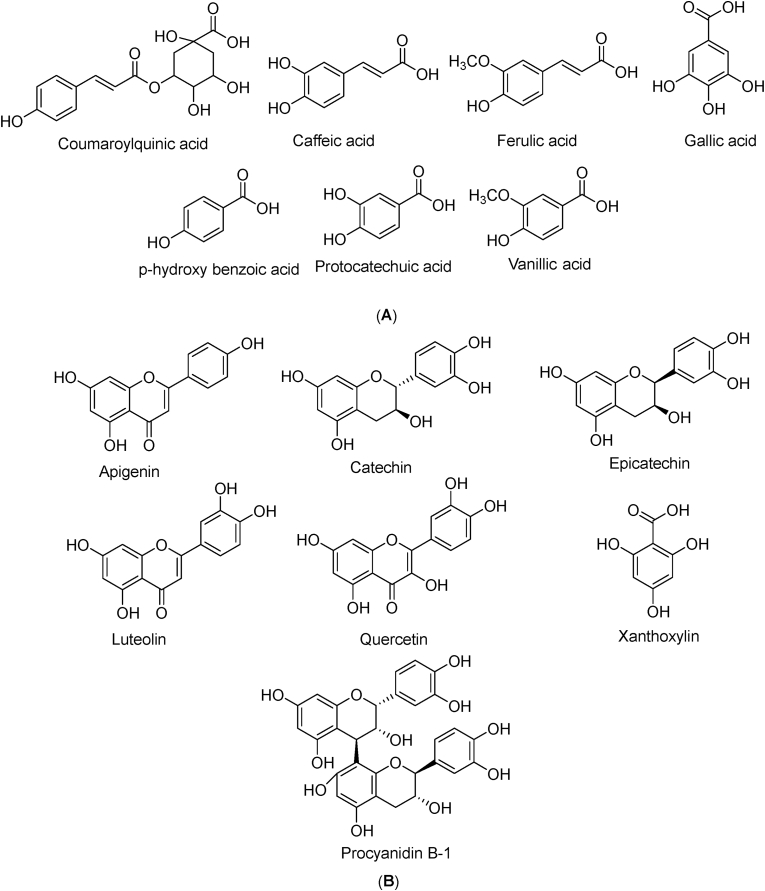
Identified different common antioxidant compounds in Ajwah, Safawy, Sukkari date palm using UPLC-QTOF-MS. In figure (A), the structures are common phenolic compounds and in figure (B), the structure of common flavonoids.

Chromatographic techniques coupled to mass spectrometer have always been desirable in analytical uses because of their high sensitivity, specificity and the ability to detect hundreds of analytes in a single run without complexity as compared to other analytical tools [54]. In our study, ethanol extracts of Ajwah, Safawy and Sukkari date cultivars were analyzed by UPLC-QTOF-MS in positive ion mode. Previous studies revealed that the fruits of date palm possess a complex chemical system comprising of amino acids, fatty acids, sugars, phenolics, flavonoids, sterols, phytoestrogens, carotenoids and vitamins, [1,30,[55], [56], [57]]. Chemical constituents reported so far from different date cultivars were matched with the molecular ion peaks observed in the mass spectrums (Fig. 3a, b, c) and the results show that these date palms contain different classes of phytoconstituents. The chemical compositions of all the three date cultivars seem to be more or less similar with some exceptions (Table 4). A total of 22 compounds were identified from the three date extracts under investigation. In addition to [M+H]^+^ peaks, we have taken [M+Na]^+^ and [M+K]^+^ peaks into consideration when analyzing the mass spectra since ESI + ionization technique can lead to sodium and potassium adduct peaks in high resolution mass spectrometry [58]. The chromatograms and magnified mass spectrums are provided in the supplementary material (Supplementary Figs. S1-S18). We revealed a number of compounds including phenolic acids, flavonoids, sterols and phytoestrogens in these date cultivars that can be related to their antioxidant potential [59]. These compounds especially the phenolic acids and the flavonoids (Fig. 4) are very common and well established dietary antioxidants with diverse health benefits [60,61]. In addition, sitosterol among the identified sterols and all of the identified phytoestrogens are reported to possess antioxidant potential [[62], [63], [64]]. Though this analysis is merely an overview of the chemistry of these dates as it was a qualitative analysis, but the difference in their chemistry can be directly correlated to their different degrees of antioxidant as well as other biological activities. In our previous study, we measured some known polyphenols in these extracts and the results are in harmony with the current study. We found that the amounts of these polyphenols present in these date cultivars are in the order of Ajwah > Safawy > Sukkari [15]. Thus, this result also supports the antioxidant potential of these date cultivars as well as their degree of activity.

**Table 4:** List of compounds identified in the collected dates through UPLC-QTOF-MS analysis.

Sl. No.	Group of compounds	Name of compounds	Actual Mass (M)	Identified peaks in date cultivars			Ref.
				Ajwah date	Safawy date	Sukkari date	
1	Phenolic acids	Coumaroylquinic acid	338.309		[M+ Na] ^+^		[1]
2		Caffeic acid	180.157		[M+H] ^+^	[M+H] ^+^	[30]
3		Ferulic acid	194.184	[M+H] ^+^	[M+H] ^+^	[M+H] ^+^	[1]
4		Gallic acid	170.119	[M+ Na] ^+^		[M+ Na] ^+^	[30]
5		*p*-Hydroxy benzoic acid	138.12	[M+K] ^+^	[M+H] ^+^	[M+H] ^+^	[30]
6		Protocatechuic acid	154.12	[M+H] ^+^	[M+H] ^+^	[M+H] ^+^	[30]
7		Vanillic acid	168.146	[M+ Na] ^+^			[57]
8	Flavonoids	Apigenin	270.236	[M+K] ^+^	[M+K]^+^	[M+K]^+^	[30]
9		Catechin	290.27	[M+ Na] ^+^	[M+ Na] ^+^		[30]
10		Epicatechin	290.27	[M+ Na] ^+^	[M+ Na] ^+^		[30]
11		Luteolin	286.236		[M+ Na] ^+^		[30]
12		Procyanidin B-1	578.52	[M+H] ^+^	[M+H] ^+^	[M+H] ^+^	[30]
13		Quercetin	302.235	[M+ Na] ^+^	[M+ Na] ^+^	[M+H] ^+^	[30]
14		Xanthoxylin	196.199		[M+ K] ^+^		[1]
15	Sterols	Campesterol	400.68	[M+H] ^+^	[M+H] ^+^	[M+H] ^+^	[30]
16		Isofucosterol	412.69		[M+H] ^+^		[30]
17		β-Sitosterol	414.72			[M+H] ^+^	[30]
18	Phytoestrogens	Genistein	270.236	[M+K] ^+^	[M+K]^+^	[M+K]^+^	[30]
19		Lariciresinol	360.401	[M+ Na] ^+^	[M+H] ^+^	[M+ Na] ^+^	[30]
20		Matairesinol	358.385	[M+ Na] ^+^		[M+ Na] ^+^	[30]
21		Pinoresinol	358.385	[M+ Na] ^+^		[M+ Na] ^+^	[30]
22		Secoisolariciresinol	362.416	[M+K] ^+^	[M+ Na] ^+^	[M+K]^+^	[30]

## 4 Conclusion

Present investigation confirmed that Ajwa, Sukkari and Safawy dates possess potential antioxidant activities that have a strong correlation with their phenolic, flavonoid and tannin contents since these phytoconstituents are the main antioxidant principles found in natural sources. These dates also contain different bioactive constituents including different phenolic acids, flavonoids, sterols and phytoestrogens, which can be partly responsible for their antioxidant activity. Therefore, these fruits can be good source natural antioxidant and can prevent a number of ailments.

## Declaration of competing interest

The authors declare that they have no competing interests.

## Abbreviations

DNA: deoxy ribonucleic acid
DPPH: 2, 2-diphenyl-1-picryl hydrazyl
GAE: gallic acid equivalent
IC_50_: inhibitory concentration 50%
QE: quercetin equivalent
ROS: reactive oxygen species
TPC: total phenolic content
TFC: total flavonoid content
TTC: total tannin content
UPLC-QTof-MS: ultra pressure liquid chromatography - quadropole time of flight mass spectrometry
TAC: total antioxidant capacity
UPLC: ultra pressure liquid chromatography
