# Supplementary material for: Antioxidant properties and phenolic profiling by UPLC-QTOF-MS of Ajwah, Safawy and Sukkari cultivars of date palm
Source: Biochem Biophys Rep. 2021 Jan 18;25:100909. doi: 10.1016/j.bbrep.2021.100909 (PMC7820033; doi:10.1016/j.bbrep.2021.100909)
Supplement: Multimedia component 1 [file mmc1.docx]

**Supplementary material**

**Antioxidant Properties and Phenolic Profiling by UPLC-QTOF-MS of Ajwah, Safawy and Sukkari Cultivars of Date Palm**

**Short Title: Antioxidant properties and phenolic profile of three date cultivars.**

S. M. Neamul Kabir Zihad^1^, Shaikh Jamal Uddin^1,*^, Nazifa Sifat^1^, Farhana Lovely^1^, Razina Rouf^2^, Jamil A Shilpi^1^, BassemYousef Sheikh^3^, Ulf Göransson^4,5^

*^1^Pharmacy Discipline, Life Science School, Khulna University, Khulna 9208, Bangladesh*

*^2^Department of Pharmacy, Faculty of Life Science, Bangabandhu Sheikh Mujibur Rahman Science and Technology University, Gopalganj 8100, Bangladesh*

*^3^College of Medicine, Taibah University, PO Box 456, Almadinah Almunawarah 41411, Saudi Arabia*

*^4^Division of Pharmacognosy, Uppsala University, Biomedical Center, Uppsala, SE, 75123, Sweden.*

*^5^Department of Medicinal Chemistry, Uppsala University, Biomedical Center, Box 574, Uppsala, SE, 75123, Sweden.*

***Correspondence:**

**Prof. Dr. Shaikh Jamal Uddin**

E-mail: uddinsj@yahoo.com

Telephone: +8801711337375

Fax: +880-41-731244

ORCID ID: 0000-0003-3163-2118


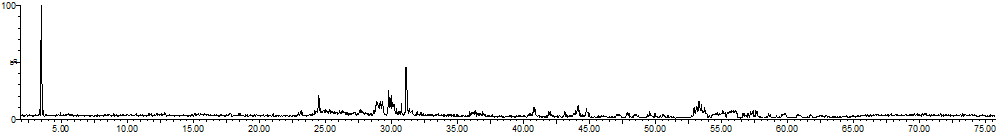


**Figure S1** UPLC chromatogram of Ajwah date extract.


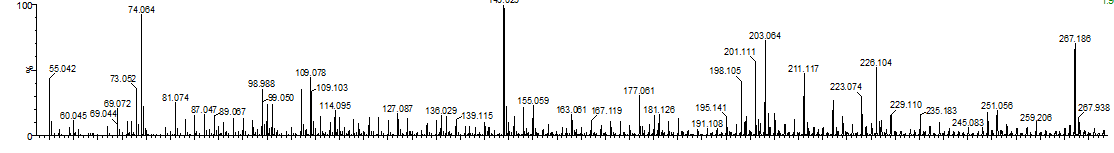
**Figure S2** Magnified peaks of mass spectrum of Ajwah date extract with mass range 0-275 m/z.

C7

C6

C5

C4

C3


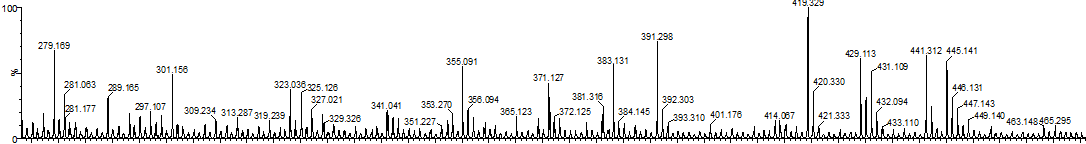
**Figure S3** Magnified peaks of Mass spectrum of Ajwah date extract with mass range 275-480 m/z.
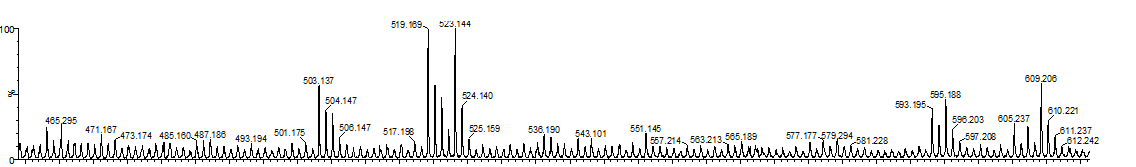
**Figure S4** Magnified peaks of Mass spectrum of Ajwah date extract with mass range 480-620m/z.
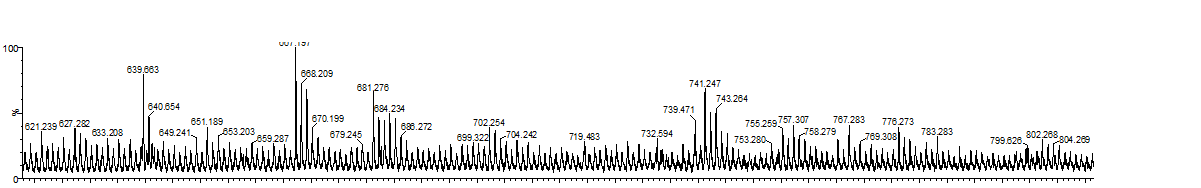
**Figure S5** Magnified peaks of Mass spectrum of Ajwah date extract with mass range 620-810m/z.
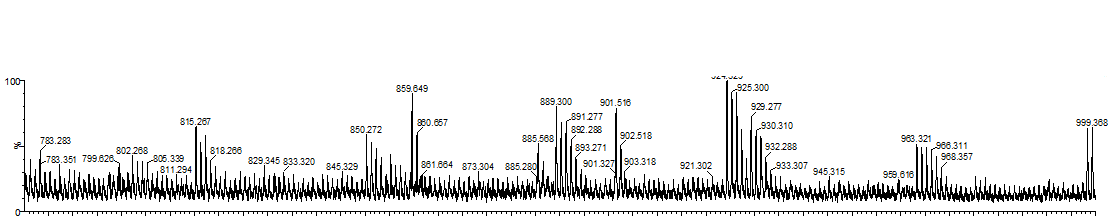
**Figure S6** Magnified peaks of Mass spectrum of Ajwah date extract with mass range 780-1000m/z.

C12

C20,C21

C19

C15,C22

C13

C8,C18

C9,C10


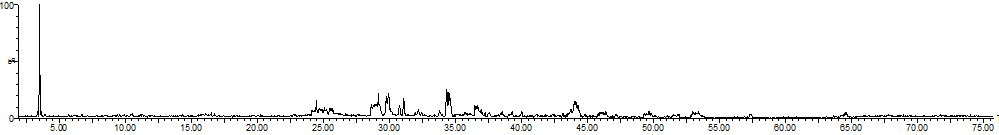


**Figure S7** UPLC chromatogram of Safawy date extract.


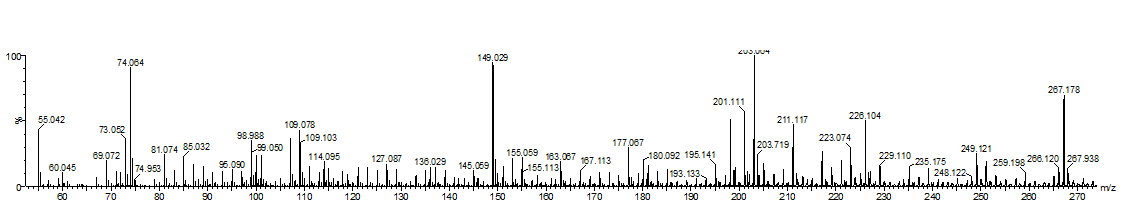


C2

C6

C5

C14

C3

**Figure S8** Magnified peaks of Mass spectrum of Safawy date extract with mass range 0-275m/z.

C1,C19

C13

C9,C10


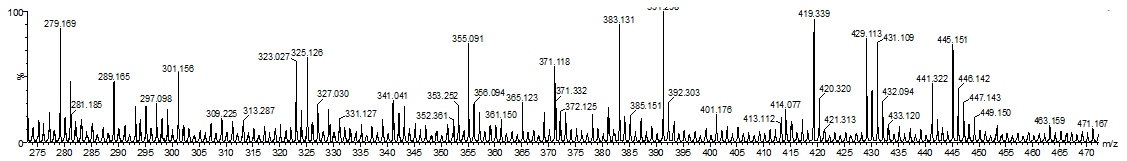
**Figure S9** Magnified peaks of Mass spectrum of Safawy date extract with mass range 275-480m/z.

C16

C15

C22

C8,C11,C18


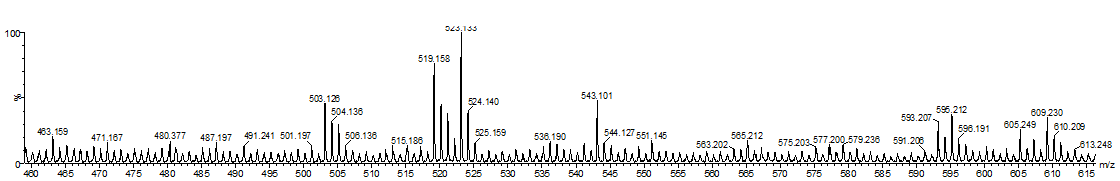
**Figure S10** Magnified peaks of Mass spectrum of Safawy date extract with mass range 480-620m/z.
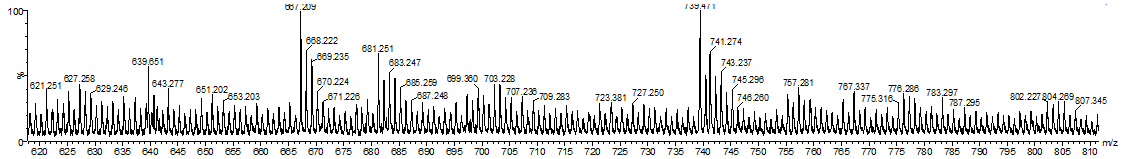
**Figure S11** Magnified peaks of Mass spectrum of Safawy date extract with mass range 620-810m/z.
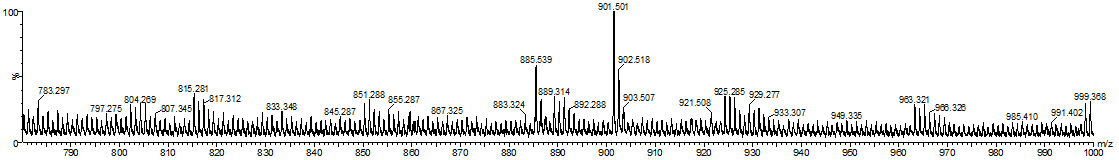
**Figure S12** Magnified peaks of Mass spectrum of Safawy date extract with mass range 780-1000m/z.

C12


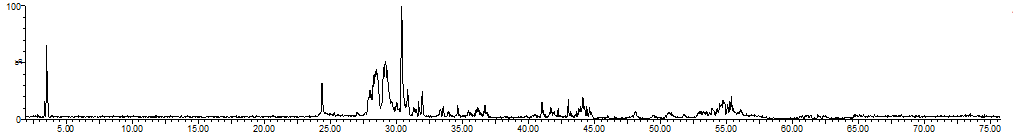


**Figure S13** UPLC chromatogram of Sukkari date extract.

**
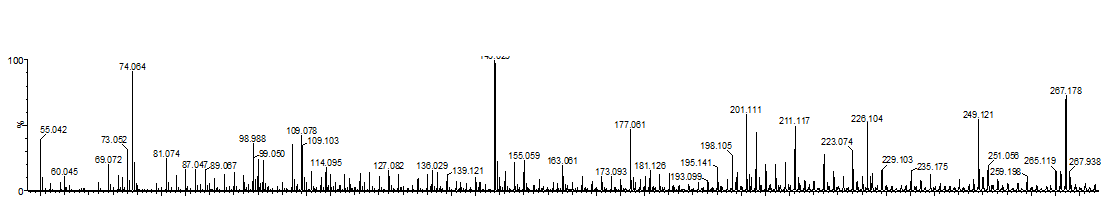
Figure S14** Magnified peaks of Mass spectrum of Sukkari date extract with mass range 0-275 m/z.

C3

C15,C22

C20,C21

C4

C2

C6

C5

**
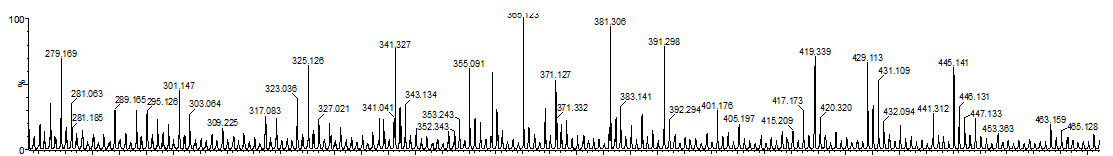
Figure S15** Magnified peaks of Mass spectrum of Sukkari date extract with mass range 275-480m/z. **
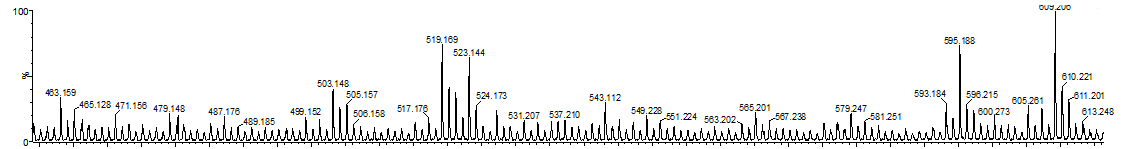
Figure S16** Magnified peaks of Mass spectrum of Sukkari date extract with mass range 480-620 m/z. **
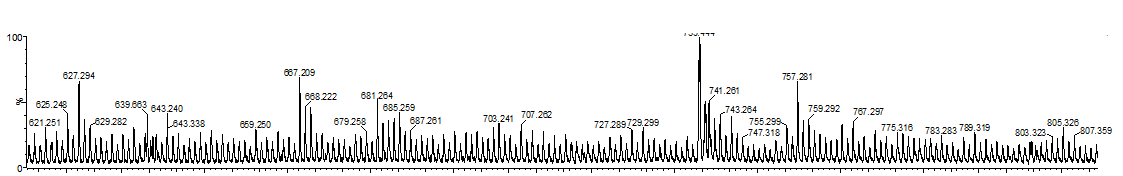
Figure S17** Magnified peaks of Mass spectrum of Sukkari date extract with mass range 620-810m/z.**
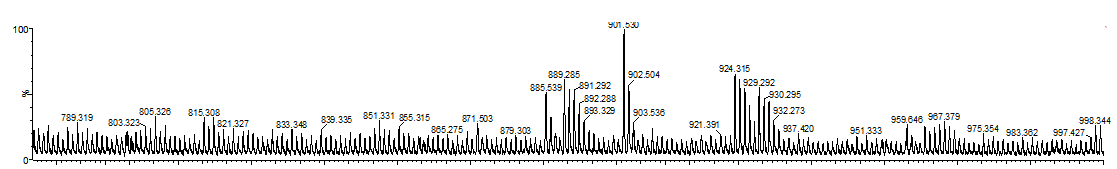
**

C19

C17

C8,C18

C13

**Figure S18** Magnified peaks of Mass spectrum of Sukkari date extract with mass range 780-1000m/z.

C12
